# The Editor's Letter-Box

**Published:** 1917-04-07

**Authors:** L. Edward Williams

**Affiliations:** 229 Boundary Street, Liverpool


Sir,—As bearing on the discussion of manipulative surgery and Mr. H. A. Barker's success therein, perhaps you would like to publish the particulars, written by the patient, of the following case, the accuracy of which I can vouch for: "Flat feet and with adhesions, partly caused by inflammation at time of scarlet fever. After consulting three doctors was told by them there was no cure, but to wear supports, except one, who told me to do exercise for them. I wore the supports for ten years, receiving no benefit whatever, and could not walk at all without great pain. I was then taken to Mr. Barker, who said at once he could completely cure them, and in about a dozen visits, without any anæsthetic, he did so. This happened twelve years ago. I have never had the slightest trouble since my last visit, and have been for ten-mile walks." After all, manipulative operations are only a branch of surgery, and any methods or ideas from an intuitive and successful manipulator outside the profession should be welcomed in true Pauline precept—"Prove all things: hold fast that which is good."—Yours truly,

L. Edward Williams, M.R.C.S.E.

229 Boundary Street, Liverpool,
April 2, 1917.

---

*** Mr. Barker has submitted to us a great many letters that have appeared in the *Medical Press*, many of them written by qualified medical men, and the rest by men of position, whose testimony we do not impugn. From this testimony it appears *primâ facie* that Mr. Barker does possess special skill in reducing dislocation of the semilunar cartilage of the knee, and that he has had successes in other injuries of joints. This we do not doubt, and this we have not questioned. At the same time, the testimony he adduces does not in the least traverse the position we have taken up. We never denied and never doubted that Mr. Barker has had successes, and striking successes. What we drew attention to is that we do not hear of his failures, and that the application of violent methods to injured limbs, if it is not successful, may be disastrous. Nothing in the correspondence we have perused militates in the least against this conclusion. We understand from a leading article in the *Medical Press* that Mr. Barker is willing and anxious to demonstrate and explain his methods to a competent committee of surgeons, provided the committee will draw up and issue a report on the efficacy and curative value of his methods. This seems on the face of it a fair offer, but there is an objection to it which would very likely not occur to a non-medical person, but which is a very grave objection to a medical man. Such a report, if favourable, and as far as it was favourable, could be used by Mr. Barker for advertising purposes, with the full signatures of the members of the committee. This is a measure to which no self-respecting surgeon would consent, and which would bring him, if he did consent, within the disciplinary powers of his College, and even of the General Medical Council. It is difficult to see how this disadvantage could be obviated. No undertaking that Mr. Barker could give would bind his friends. We are quite prepared to admit that Mr. Barker's methods deserve investigation, and if he can devise an unobjectionable method by which they can be investigated we shall be prepared to support his effort; but the methods that his supporters have pursued are not such as to command our sympathy.—Ed. The Hospital.

